# Dilemma and countermeasure of sustainable leadership in physical education development in southern rural Ningxia, China

**DOI:** 10.3389/fpsyg.2022.947694

**Published:** 2022-08-29

**Authors:** Xiaoya Fu, Weiqiang Zhu

**Affiliations:** ^1^College of Physical Education and Health, East China Normal University, Shanghai, China; ^2^College of Physical Education, Ningxia Normal University, Guyuan, Ningxia, China

**Keywords:** sustainable leadership, sustainable strategies, sporting leadership, technological support, physical education

## Abstract

Globally, above 1.4 billion adults did not reach the recommended level of physical education in their daily life, thus triple intendent reforms are proposed by the Ministry of Physical Education for the development of sporting leadership in schools, colleges, and universities, which are essentially important for the development of physical and mental health of the students. This article analyzes the situation of lacking sustainable sporting leadership among other factors related to Physical Education (PE) resources in the southern areas of Ningxia. A mixed and multi-method approach was adopted to conduct the study. First, an in-depth but an open-ended qualitative interview with the professionals was carried out, followed by cross-sectional data collected from the respondents in two districts of the southern mountainous area of Ningxia. Moreover, a case study was included to support the phenomenon from a contextual perspective. The study present that PE education needs a modernization and rejuvenation plan to link with PE development and its sustainable execution and implementation for the physical and mental development of the learners. Moreover, it is suggested to strengthen the development of physical education with/through the fields of regional integration of educational resources sharing, cultural elements and integration, latest technological tools, research-based and cultural supported curriculum, and endogenous strength construction to promote the development of school physical education. Furthermore, continuous monitoring and evaluation mechanisms need to be adopted to develop physical education in the region.

## Introduction

China has the largest education system in the world (Jin, 2013); however, the growing concern about obesity worldwide and among school children is alarming. Therefore, Healthy China 2030 and the National Fitness Program are among the triple intendent reform policies that emphasize on physical education development to meet the basic criteria for the children and adults in educational institutions. The key pillar of the educational curriculum in China is moral, physical, intellectual, and aesthetic development, and less attention has been given to the physical development program through sustainable leadership (Wang and Wang, 2020). Physical education has secured a special place since triple independent reforms were proposed by the Ministry of Education in the “Physical Education Curriculum Outline” for all the learning institutes to develop sporting leadership and more focus on physical education development in the educational institutions (Wang and Wang, 2020; Zhang et al., 2020; Xilin, 2021). World Health Organization [WHO] (2019) reported about 1.4 billion adults do not have access to the physical education facilities which is causing obesity among the school children and adults and this casuing alarming health issue among the adults reaching alarming level; the reason for the slow global progress in increasing physical activity is largely due to the lack of awareness and investment on the PE facilities especially sporting leadership.

The aim of implementing sporting leadership in education is to motivate the interest of students toward physical education and encourage the student to optimize the physical education resources in learning institutions. However, the serious problems of students’ physical health levels have not been solved and overcome. The formal curriculum was developed according to the psychological and developmental needs of the learners. Meanwhile, sports competitions and tournaments were conducted that further explored PE and added a variety of scientific and traditional games to the lockets of PE (Jin, 2013; Peng et al., 2014; Zhang et al., 2020).

Physical Education (PE) has a long history in academia and enjoyed support from the culture around the globe. In almost every culture of the world, modern or traditional, athletes are considered holistic, heroic, encouraging, pragmatic, and optimistic individuals (Jenkinson and Benson, 2010; Peng et al., 2014). There is lack of literature studies on the relaitonship between culture and physical education however, it is evident from the differences in practices, shape, context, intensity, curriculum, environment, subject matters, and social and emotional attachment with different games in different areas (Xilin, 2021). Moreover, PE was acceptable around the globe because it followed the principles of naturalism liberalism, realism, existentialism, verisimilitude, and verismo (Shi and Zainuddin, 2020). The history of PE started almost in 1820, when school started focusing on hygiene training, care development, and gymnastics for mental and physical growth. By 1950, over 400 institutions in China formally started physical education (Jenkinson and Benson, 2010; Peng et al., 2014; Li, 2021). School and college students in western countries were encouraged to participate in formal PE activities and formal games like football, volleyball, and other traditional, especially internal games, which also helped prepare learners for civil actions and defense (Zhang et al., 2020). School, college, and university students were given the stimulus to join hands in Athletic events at the start of the twentieth century (Zhang et al., 2020; Li, 2021). Educational institutions have initiated sports competitions in school and college and therefore required physical educational leaders who will handle to prepare the students for the sports and train them according to the PE activities (Lei et al., 2020). Colleges took great pride in their athletic programs, and sports scholarships became a norm. There was also a surge in people who enrolled in sports education programs to meet the growing demand for professionals in the field (Kirk, 1999; Liang et al., 2005; Quennerstedt, 2019).

PE training of the student is always complex in nature and other studies’ pressure on the students pushed them to least accept the PE training classes (Gleddie and Morgan, 2021). The modernization and mechanization of education pushed education toward competition, mental development, and mental mapping, and very little concentration is being paid to the physical development of the learners (Maksymchuk et al., 2018; Lei et al., 2020). This is evident from the shrinking of the educational institutions’ size, shifting to the highly populated and dense areas in tall buildings without proper playgrounds. In the earlier days, indoor game facilities were provided, but that is also vanishing (Ni et al., 2020).

PE was ignored and given less attention compared to other subjects in China (Quennerstedt, 2019). The policymakers forgot that only a healthy body can have a healthy mind. There is a close liaison, coordination, mutual consistency, and computability between body and mind; both synergies complement each other. They cannot perform better without each other’s support (Hector and Salinitri, 2020). The isolation of body and mind brings social, moral, psychological, and emotional dismay to the learners, which not only disturbs their current activities and studies but has a long-lasting effect on their practical lives (Koç, 2017). The non-availability of sports practices and facilities has developed a dilemma in education, which is not now limited to the densely populated urban areas but has also greatly affected rural regions around the globe. The same has been portrayed from the practices in Ningxia of China, where educational institutions have been suffering from the physical and sports facilities, which are impacting learners’ cognitive and physical abilities, however sporting leadership and goverment policies are lacking for the sustainable development of the physical education infrastructure in Ningxia (Liu and Li, 2017; Chang et al., 2021). Therefore, this article focused on the continuously decreasing state of physical education in the rural area of Ningxia of China. Thus, the main research question for this study is that “What is the dilemma of the sustainable physical education leadership in the rural areas of China with a specific focus on Ningxia?” A pilot study was conducted in early 2018 in Hubei, Ningxia on a small scale to understand the dilemma of the sporting facilities in the school, and it found that rural school sports imbalance in terms of human resources, fund unavailability, material, poor financial and economic conditions, and other facilities development deficiencies. Liu and Li (2017) highlighted the major cause of PE failure in school is the lack of policies for the sporting leaders and facilities in educational institutions. Similarly, lack of teacher development training, unavailability of the PE teacher, shrinking of the size of school and faculty, and the unclear vision of teaching and management pushed PE to the wall. Koç (2017) reported that the lack of PE teacher training and the lower pay scale of the PE teacher make PE unattractive. Furthermore, a significant cause of the damage to PE is that its evaluation is not included in the overall learners’ or schools’ evaluation, which vanishes the interest of the learner, parents, and faculty in PE, also PE teachers or leaders are not supported like the science subject teachers are supported in the country. The values of non-examination-oriented education are impossible, which has created a state of hopelessness and contradiction in learners, faculty, and students. Their self-esteem and self-confidence are seriously undermined, and their sense of self-efficacy remains very low. The values of examination-oriented education are deeply embedded in the minds of all stakeholders, which is a sizeable gap between self-existence and development and innovation. The findings of the pilot case study were also the source of motivation for the study to explore the phenomenon of physical education in the said region of China. This manuscript proposes a holistic framework for physical education development. In the ever-changing organizational environment, the needs and demands for physical education also change. This manuscript provides a continuous development model for the school, integrating education stakeholders. However, the study was limited only to the Ningxia province, and for getting more comprehensive and generalized results, the study may be incorporated into other regions and provinces of the country.

## Literature review

Physical education in China is introduced in school and become like a “Western Art” during the opium war; however, later military style PE has initiated in learning institutions (Sáez de Ocáriz and Lavega-Burgués, 2020). Military-based training in education was appreciated, and the same kind of activity was started in all formal and non-formal schools (Zhang and Bray, 2018). This movement, which was named “western art,” and westernization positively affected the physical education system and tried to modernize and align it with the latest technological tools, techniques, and models (Liu and Li, 2017; Sáez de Ocáriz and Lavega-Burgués, 2020).

Educationists and PE practitioners and experts devised new methods and models for PE development (Knox, 2020). In the 1950s, western countries and the United States initiated PE classes in school for a better healthy lifestyle promotion among the students and also to develop their physical health condition to unburden mental stress (Liang et al., 2005; Wang, 2019). This gives birth to different sports clubs’ development, which played a crucial role in PE development. In 1904, the Qing government issued an instruction to develop a structured curriculum for PE development (Quennerstedt, 2019). In the same way, during Xinhai Revolution, in 1902 and 1903, the government of the Republic of China reformed the PE curriculum, started PE classes at all levels, and aligned them with the culture and history of China, which bolstered the PE in the region (Zhao and Sun, 2020; Meng et al., 2021). PE education was gradually reformed, gymnastics was changed to PE, and comparatively less concentration was given to PE (Zhang et al., 2021).

The main reason was the harmonization of the different tribes, which led the focus more on mental development than physical development (Liu and Li, 2017). PE was changed more to sports instead of Militarism, which was widely accepted in China. In addition, the Kuomintang (KMT) Government strengthened the school management to promote PE, issued laws concerning PE, and tried to develop PE systematically regularly (Hastie et al., 2020). In the era of the foundation of the Republic of China, the reforms for sustainable PE were developed, keeping into consideration international standards. At the beginning of the foundation of the People’s Republic of China, Chairman Mao developed guidelines for PE with the slogan “To be health is in the first position, and to study is the second” and “Be in good health, study well and work well” (Liu and Li, 2017). Meanwhile, coordination was developed with different international courtiers to develop the PE; especially, agreements were signed with Russia and other Asian countries for PE development to follow the best models for PE development.

They also tried to model PE with the latest technological support besides giving social and cultural support (Jenkinson and Benson, 2010; Isidori et al., 2016). The expected outcome of the reform was that PE meets social and cultural demands besides the competence development of the learners (Lei et al., 2020). Kirk (1999) stated that education reforms in urban cities are more systematically implemented than in rural areas; additionally, the Education department and general administration are more proactive in the urban cities compared to the rural districts of China. The growing economy of the country and spending more on the education and development is the main goal he China’s government (Zhao and Sun, 2020). The reformers tried to align it with local culture and tried to develop them independently. They started investing in PE, for the students, and they tried to create better control and management mechanism for PE (Knox, 2020; Gleddie and Morgan, 2021). Besides the more recently proactive implementation of the educational reforms and promotion of physical health among school students, PE is overlooked in urban cities (Qian et al., 2018). In the reform policy implementation, leadership in the sports and physical education expert body are developed to improve the quality of PE at the school level and in the urban area. China has successfully improved the PE quality; however, the lack of facilities and leadership in sports was reported by Maksymchuk et al. (2018) and Qian et al. (2018).

### Contextual analysis of physical education in China

**FIGURE 1:**
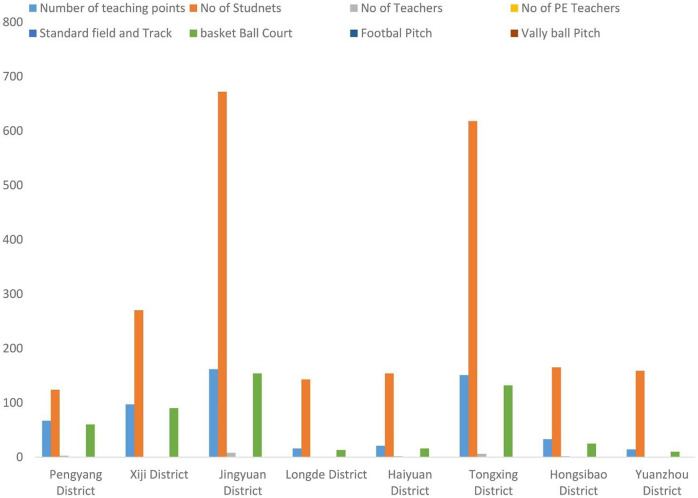
Status of non-availability of PE facilities (Source: author’s pilot data).

In 2011, the Ministry of Education promulgated the concept of “Health First” and made it an integral part of compulsory education (Hector and Salinitri, 2020; Meng et al., 2021). They promoted the teaching of Cheng, which enables students to master motor skills, develop physical fitness, gradually form a sense of health and safety and a good way of life, and promote students’ physical and mental coordination and all-around development (Zheng and Liu, 2018). However, schools in remote areas are affected by economic, cultural, transportation, and teachers, and the implementation of the physical education curriculum did not remain optimistic. Hastie et al. (2020) found that about 85% of the schools failed to complete the prescribed hours (2–3 h per week) of physical education and health curriculum in their schools in Southern Ningxia. Moreover, teaching content and sports program arrangements were limited in variety and could not meet the developmental, physical, and psychological needs of the learners (Chen et al., 2020). In the same way, implementation of curriculum integration was found different due to differences in the modules and models, which further caused curriculum evaluation, teaching evaluation, and student learning evaluation difficult and were not exercised on a scientific and empirical basis (Peng et al., 2014; Chen et al., 2020). Furthermore, a severe shortage of physical education teachers, lack of physical education facilities in schools, and reduced support from the locals and government side worsen the PE situations in the far-flung rural areas of China, which are outlined in Figure 1.

**FIGURE 2:**
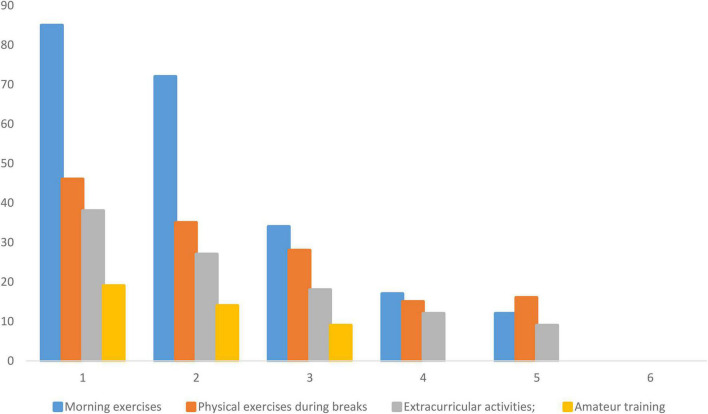
Sports schedule implementation in schools (author’s pilot data).

Li et al. (2018) documented the non-availability of the PE facilities district vise. Figure 1 indicates that in all districts, enough students were found in the school, but the number of the general education teacher is very less and none of the mentioned schools have a single professional PE education teacher, which is the main cause of reduced physical education. Similarly, except for a basketball pitch, none of the schools possess a volleyball or football ground. Moreover, only two schools have small tracks and fields for sports activity. Likewise, Figure 2 shows the practice of PE in the southern regions of Ningxia, which indicates that very rarely sports and healthy activities were performed in the schools.

Figure 2 indicates that rarely physical activities were carried out in the rural areas and the number of repetitions was very small as compared to standards. It also indicates that amateur training was not repeated during the whole week, whereas the values for morning exercise, exercise during breaks, and extracurricular activities were not encouraging, which are the main causes of the poor physical education status in rural areas of Southern Ningxia, even after the strong recommendations of the different governmental bodies and public agencies. Keeping into consideration the serious deficiencies in extracurricular physical exercise, after-school training and competitions were proposed in 2007 by CPC Central Committee and the State Council on Strengthening Youth Physical Education and Strengthening Young People’s Physical Fitness, which include but are not limited to the following criteria that students need to ensure at least 1-h practice per day; the school needs to organize group activities, but these were hardly carried (Knox, 2020; Wang and Wang, 2020). Moreover, during the pilot investigation, it was observed that out of 180 rural teaching points, only 35% of the schools were able to fully implement the school sports system, which shows the lack of sportsman agents, supervision, and interest, which were also highlighted by previous researches (Peng et al., 2014; Chen et al., 2020).

### Models and theories of physical education

There can be many models of PE, enclosed in social, cultural, and scientific stratums, but five (5) of them are comprehensively followed, accepted, and practiced around the globe. These models are providing a variety of ways and means to implement PE in the best possible, feasible, optimal, and suitable ways (Tsai and Zhou, 2017; Hastie et al., 2020). Moreover, these models are simple to practice and have closer liaison with the local culture, therefore they are accepted in all localities, where they are implemented with little variations by Wang and Hu (2017) and Hector and Salinitri (2020). They can diversely deliver physical education lessons to provide essential skills to the varying number of students’ challenges, besides preparing them for future challenges (Liu and Li, 2017).

a. **Traditional Model:** In this model, the teacher generally decides on the elements of the game, and the students are moved to the practice (Maksymchuk et al., 2018; Quennerstedt, 2019). On best performance, students are rewarded, but this model is criticized because it is snatching students’ liberty and giving more power to teachers for decision-making (Zhang et al., 2020). However, still it has utility for developing PE in rural and economically privileged areas, as it is less costly, has traditional and cultural support, and can be practiced after school hours and within boundaries (Hector and Salinitri, 2020; Wang and Wang, 2020).
b. **Teaching Games for Understanding (TGfU):** In this model, students play the game for improving skills with the hope to perform better (Quennerstedt, 2019).
c. This approach is considered better as the learner enjoys freedom; however, fewer directions, training, and advice are involved, where the activity may not take place in a well-planned scientific manner and may not cultivate full benefits from it (Liu and Li, 2017; Wang, 2019; Knox, 2020).
d. **Game Sense:** It emphasizes more on developing good decision-making among players while involving them in the decision-making process and different game puzzles, which prepare students both mentally and physically (Liang et al., 2005; Maksymchuk et al., 2018; Hector and Salinitri, 2020).
e. **Cooperative Learning:** It is a peer learning model, where sportsmen have to groom themselves besides taking care of their mates (Tsai and Zhou, 2017). It is considered less participative regarding the layout of the lesson, but develop the confidence of the learner through student-centered activities through “Jigsaw” like practices where student participate and compete for a cause (Liu and Li, 2017; Hastie et al., 2020).
f. **Sport Education:** The Sport Education model is unique and considered the best where students focus on their learning through mini-learning activities (Kirk, 1999). Students adopt roles according to their interests and inclination and compete in a more traditional tournament for exhibiting their skills and for winning the models and games (Zhang et al., 2018).

### Educational theoretical models to support and promote effective physical education and its pedagogy

#### Constructivism

The theory of constructivism asks students and teachers for developing and connecting ideas and practices regarding sports and PE inside and outside the classrooms (Zhang, 2017; Vuong, 2022). It encourages creating meanings and values for self and society. They operationalized Vygotsky’s principles of social construction for meaning, scaffolding, and inquiry-based learning. It consists of the following elements:

a. **Active learning** involves learners in active decision-making processes, developing their critical thinking, contextualizing problems and solutions, and giving learners freedom and support for planning and gaming (Jenkinson and Benson, 2010; Jin, 2013).
b. **Social Learning** asks for devising invasion-based games and then in pairs, working as coaches to provide feedback (Pengli, 2019). It helps in constructing individual and social interaction and in the same way forming and testing their knowledge for self and social development (Qian et al., 2018; Vuong, 2022).
c. **Creative learning** supports imagination, creativity, and ownership of the knowledge developed (Peng et al., 2014). Students work in a collaborative environment and develop and criticize one another’s tasking and games, which are elemental mental and physical functions of the sport. It develops higher-order critical thinking and skills to enhance sports learning (Gao and Ren, 2019). These activities engage students in creating thought through an “active linguistic and cognitive response,” which also promotes constructive pedagogy for classroom use. This concept works not only in an attempt to extend students, but also to provide a supportive learning environment (Qiu, 2019).

### Inquiry-based learning and guided discovery models achieving an effective physical education delivery

Like constructivism, inquiry-based and guided discovery models and theories can be utilized for student motivations for effective motor skills and cognitive development. According to inquiry-based learning, students deeply and authentically develop their skills, when they are presented with different problems, external stimuli, and physical excisions (Qian et al., 2018). It promotes skill-based learning through practice reflection, and open-ended, complex, and thoughtful scenarios, which inspire deep learning. In PE it helps in developing different ball games, ball choices, movement styles, running, kicking, throwing, etc. with mental and physical freedom, which helps in boosting all inner and hidden talents of the athlete through a variety of responses (Liang et al., 2005; Peng et al., 2014). Tsai and Zhou (2017) suggested a guided discovery approach for “movement solutions” with productive pedagogical support from the teacher. This process will also help in accommodation and assimilation through recalling their previous knowledge and practices (Qiu, 2019).

In the same way, Teaching Games Understanding (TGfU) supports higher-order process thinking in PE. Qiu (2019) and Knox (2020) recommended that although it may be difficult for developing higher-order skills in PE, TGfU can help the teacher tactically take bold decisions during games besides adding to their enjoyment in games while developing difficult and higher-order targets. Furthermore, Wang and Wang (2020) acknowledged that TGfU helps in developing a holistic personality, promotes enjoyment and student-centered learning, develops varying abilities, and makes the learner more efficient in implementing mental and physical tasking. According to research, PE teachers need to engage the learner in quality enjoyable learning opportunities for better outcomes (Qiu, 2019). They need to develop an experimental learning environment, relevant to health literacy skills (Maksymchuk et al., 2018; Zhou et al., 2018). TGfU is an effective strategy that would foster such considerations and promote and sustain student cooperation, encouragement, and collaboration (Chen et al., 2020; Shi and Zainuddin, 2020). It will encourage ongoing participation in PE, will get parental support, and will reap positive and beneficial health outcomes. Research has shown that TGfU has resulted in improved fundamental movement skills for students, and students who are competent in fundamental movement skills are more likely to enjoy sports and activities and develop a lifelong commitment to physical activity (Peng et al., 2014; Shi and Zainuddin, 2020). The best application of the TGfU model is that it is equally applicable to male and female and minimize feelings of incompetence and a feeling of being undervalued among different strata of society and population (Pengli, 2019). Moreover, the TGfU model encourages decision-making skills among learners in all kinds of games, maybe target games, net/wall games, striking/fielding, or invasion games. One of the beauties of the TGfU is its student-centeredness, freedom, student appreciation, and creation of high-order skilling, which is also the focus and recommendation of constructivism (Tsai and Zhou, 2017; Qian et al., 2018). In conclusion, it believes in the following:

I. Access, evaluate, and synthesize information to take action and protect, enhance and promote their own and others’ health.
II. It helps in developing social, moral, behavioral, and cognitive skills and promotes personal and social cohesiveness and development.
III. It believes in the acquisition, application, and evaluation of motorcycle skills and respectful relationships among body, mind, oneself, and society.
IV. Engage and enjoy regular movement-based learning.

Therefore, in developing a holistic, balanced, and motivated child, many departments, elements, and stakeholders are supposed to join hands together. Based on the content analysis, child development is an iterative and interactive process.

**FIGURE 3:**
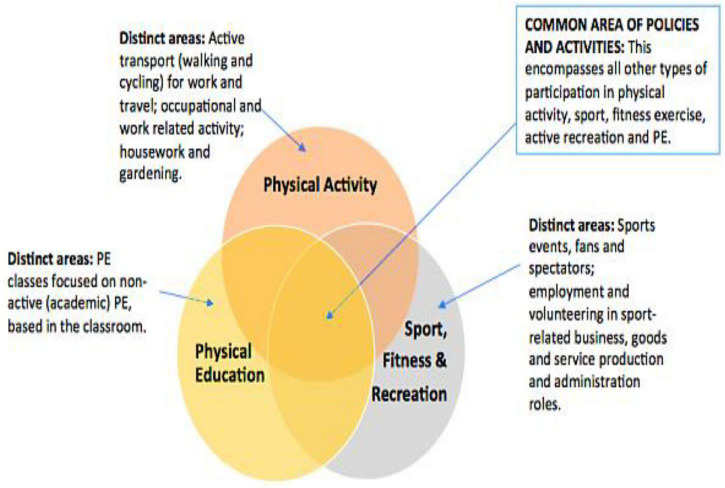
Physical education development model (author’s proposed).

After a comprehensive review of the literature, pilot, and contextual studies, the study came up with the following comprehensive model for holistic PE development as shown in Figure 3: sports leadership, sustainable goal-based PE, student readiness, social and cultural support, government, and other social and stakeholder support and encouragement. Moreover, it also talks about the role of models and theories of educational and physical development, their comprehension, and implementation for better PE development. Furthermore, due to social, cultural, and technological support, it is cost-effective to be implemented, which the China government needs to implement for PE development. In the same ways, the policymakers have developed a mechanism for continuous curriculum development, diagnosis, and testing.

### Research design

Based on the objectives of the study, the multi-stage mixed method was applied to explore and comprehend the underlying phenomena in detail. In the first stage as shown in Table 1, content analysis was done besides contextual analysis to get the ground information of the PE in the mountainous and rural areas of Southern NingXia. In the second phase, a pilot study was conducted to explore the underlying state of PE. Based on the pilot study results, a qualitative open-ended interview was conducted with the education experts to further clarify the stances raised in the pilot phase of the study; during the interview, the objective of the interview was explained verbally and consent was scored. In the final stage, from the reliability and generalizability standpoint, a quantitative, cross-sectional large-scale study was conducted. Data were collected from the two-state of southern areas of Southern Ningxia in China using the 5-Likert scale and adopted questionnaire added in Supplementary Appendix. Moreover, to make the result more cohesive, specific, and reliable, the target population was divided into two strata, i.e., Faculty (Teachers) and Students, to get both perspectives individually and clearly. Furthermore, a convenient sampling technique was applied in the third phase of the data collection, and the reason for it was that those qualify the requirement, i.e., enrolled in school as a student and teacher, were eligible for inclusion in the study.

**TABLE 1:** Descriptive data analysis (student data).

Questions	Mean	*SD*
The outdoor areas (e.g., playground, field, facilities) at my school are in good condition.	1.344	3.16
The indoor areas (e.g., gym) at my school are good condition.	1.137	3.72
My school has enough sports equipment for students to use.	1.274	3.04
My school has sports and physical activity equipment that students can use during recess and lunchtime at school.	1.870	3.40
The gym classes at my school are long enough.	1.084	3.48
The gym classes at my school occur often enough during the week.	1.581	3.40
We do a variety of activities in a games class at school.	1.301	3.12
We have good coaches at my school.	1.382	3.64
My school offers other physical activities or organized sports for students after school.	1.377	3.16
I can find out about community physical activity and sports opportunities at my school.	1.124	3.80
Students usually encourage me to participate in sports and physical activity at school.	1.325	3.52
Students usually make negative comments when I’m being physically active at school.	1.059	4.04
Teachers usually encourage me to be physically active at school.	1.422	2.24
My game teacher is physically active with students at school.	0.988	3.32
Teachers supervise students being physically active at recess or lunch.	1.382	2.88
Teachers think physical activity is important for students at my school.	1.525	2.36
The indoor and outdoor areas at my school are safe to use.	1.377	2.84
The equipment at my school is safe to use.	1.623	3.44
The indoor and outdoor areas at my school are supervised.	4.671	3.76
Other students make me feel safe when I am physically active at school.	1.609	2.96
Valid N (list wise)		

A total of 125 students and 433 faculty members participated in the study. Convenient sampling was adopted for the study. Sample size for the faculty members were unknown therefore according to Peng et al. (2014) 384 can be the suitable sample size, however, more than 384 faculty members participated in the questionnaire survey which is higher than the cut-off value. In the student sampling, only those students were approached who participated in the sports activities from the region, therefore, the sample size shrunk. A total of 170 students interested in different games were contacted and the study received 125 complete applications after the screening processes, which comes to more than 70% and is considered a good representation of the study.

### Ethical statement

Research and Development Board of Factuality of Physical Education and Health, East China Normal University has provided written consent to collect the data with the condition no human picture will be public, and all teachers, parents, and students were verbally informed about the purpose of the study and written signed permission of the research granted by the university were present whenever it was demanded. Respondents were informed; they understood the nature of the research and volunteered for this study.

### Qualitative interview thematic analysis

In the first phase, a qualitative study was conducted as per the need and requirements of the study design. The study was conducted in February 2019 in the mountainous and rural areas of Southern NingXia. An open-ended structure questionnaire was designed on the results of the pilot study and a total of 10 experts participated in the interview, among them six were men and four were women, having bachelor’s degrees with professional (education) related experiences. The main question asked was “Do you consider that psychical education is declining in the remote areas of China?,” followed by several contextual supportive mini questions and explanations, to clarify the content clearer for understanding. The thematic analysis of the open-ended interview is given here.

Almost 100% of respondents agreed that “Yes,” “it has been declined and further declining.” The major reason explained by the respondents were “the poor state of physical instructor training and unequal distribution of the sports facilities in the mountainous and rural areas of Southern Ningxia” additionally, football, volleyball, and other ground facilities need to be developed including the governance and sport leadership.” Moreover, they added that the “non-availability of structured curriculum has reduced the attention of the students, teachers, and parents in getting physical education.” In the same way, physical education is being taken as a part-time activity, which has shifted the mind of all education stakeholders that physical education may not help in getting/developing a good career in the practical lives and may not be a source of handsome earning in future.

Similarly, respondents suggested that on the priority level, both the Central Committee and the State Council should devise policies for promoting physical education and they uttered more responsibility to the local government.

According to respondents, China’s population is too large, so for the central government, it becomes an uphill task to reach every stakeholder, therefore, major responsibilities lie with the local government. One of the best suggestions, which can be better helpful in promoting physical education, is to align it with local culture, in which besides students, parents will also take interest and it will become a community congregational activity, where the sportsmen will not be waiting for external supports and governmental packages, but locals without considering their financial position will contribute for the development of the PE. Everyone will consider it their priority to get physical education. This premise is also supported by the local teachings of China, as Chairman Mao’s theory of sports development called “sports theory” for the development of physical education through local cultural and ethical alignment.

Furthermore, they also sought the help of the university teachers in developing the state-of-the-art curriculum for physical education besides developing the training and facilitation modules and techniques for physical education. They wished to grade physical education as one of the key performance indicators (KPI) in the entrance exam to the middle or high schools/college/university level, which will promote physical education morale in remote areas.

## Results and analysis

This study’s demographics are sub-divided into student groups and teacher groups. This study reported that a convincing sample of the questionnaire was distributed among the high school students actively involved in the sports. A total of 170 students were sent the questionnaires, of which 125 respond to them. A total of 73.5% of the respondents were students and 88.60% of the respondents were teachers and experts who participated in the questionnaire survey.

Students’ background profiles show that the percentage of male respondents is 69.0% and about 31.0% are female participants, most of the respondents are in the age of 16–19 years old high school students and often participated in the school PE and sporting classes. A total of 67% claimed they face difficulties due to limited facilities compared to the students studying and living in urban areas.

In the contrary, teachers’ demographic background information shows about 57% are male teachers and 43% are female respondents, most of them are in the age of 27–33 years old and have served for more than 5 years. About 84% are from the other discipline/teaching other subjects and only 16.0% of the respondents listed are trained PE teachers, who due to lack of interest and future of the PE teacher found the least attractive option to become PE teacher, therefore they do agree with the statement of lack of interest and future opportunities oppressing them to not become PE teacher in the school.

### Quantitative analysis of the data

In the third phase of the project, as mentioned in the research design stage, data were collected from the students and teachers. Mean of the all question is low as compared to their variances, and the bigger value of variances means more distraction, deviation, and non-conformance to the needs and requirements. It simply means that students are not satisfied with the present state of PE.

The teacher’s data also portray the almost same situations as predicted by the students’ data as listed in Table 2. Availability of facilities (AF), Trained Professional Physical Teacher (TPPT), State Supports for Physical Education (SSPE), Support from Management (SM), Miscellaneous Problem of PE (MP), and Student Related Problems (SRP) all constructs have small mean values as compared to variances, which indicate that teachers are not happy from the conditions of the physical education facilities and support activities.

**TABLE 2:** Descriptive data analysis (teacher data).

	Mean	*SD*
AF	1.24605	3.3261
TPPT	1.19884	2.4522
SSPE	1.06326	2.4435
SM	1.00288	2.7000
MP	1.06869	3.7478
SRP	1.21504	3.6522
Valid N (list wise)		

Independent t-test to compare the means for PE leadership.

According to the results of the two-sample *t*-test, there is very less variation among the means of the two samples, which shows homogeneity of the samples (Table 3). Similarly, both *P*-values are less than 0.05, which indicates their significance followed by the values of Cronbach alpha. These values also proclaim the reliabilities, validity, and consistencies of the instrument. So, according to the results, the conditions of the *t*-test meet and remain valid for the study.

**TABLE 3:** Independent *t*-test.

Independent samples *t*-test					
	**Test**	**Mean**	***df***	***p***	**Cronbach alpha**
PE leadership	Student	2.365	38.00	0.003	0.748
	Faculty	2.201	35.05	0.000	0.898

## Discussion

These shortcomings of PE can be overcome, first with the construction of a sports resource sharing system in regional integrated schools, which can be easily done remarkably through information technology (Liu and Li, 2017; Vuong et al., 2021). As the concept of resource sharing is successfully comprehending other walks of life can be easily replicated and applied to PE in rural areas also (Chang et al., 2021). The study indicates the dilemma of physical education in rural areas, and it is attributable to leadership (or leaders’ mindset). Thus, it is essential to discuss how the mindset among managers, planners, policymakers, etc., is shifted to embrace the values of physical education. This mindset shift can be actualized by affecting their subjective cost-benefit judgment and beliefs (Vuong and Napier, 2015). It can be done through government actions, excellent urban professional physical education teachers’ selection, and the designing and implementation of a high-quality physical education curriculum (Jinga and Kimb, 2020; Vuong et al., 2022). Similarly, improvement of general practitioners’ training systems and scientific management can help in improving PE. Moreover, PE should be valued in the exam system and need to be evaluated for getting admission to the next grade, which will intrinsically encourage the pupils, parents, and teachers for promoting PE in rural areas. Furthermore, it may create more sporting employment by developing and strengthening the sporting facilities for students in schools which are considered indispensable for promoting PE. Besides its rich, cultural, social, and military history, China has a good value system of encouraging heretic physical activities. However, this enduring tradition has been facing challenges continuously due to its demographic and environmental changes related to urbanization and lifestyles (Wang and Wang, 2020). Due to reduced practices of PE, there are escalating levels of overweight and obesity, and decreasing levels of physical fitness in the Chinese population. This trend is continuing and still; as a nation, they do not want to get behavioral changes and the recent pandemic of COVID-19 worsen the situation. This trend has adversely affected general public health and the study of Physical Activity and Fitness in China—The Youth Study (PAFCTYS) has given alarming results, which are consistent with the results of this study. High prevalence of sedentary behaviors and unhealthy weight, and low levels of physical fitness among school-aged children have led the whole generation toward an unhealthy life (MingChang and HuaJie, 2013; Wang, 2019; Wu et al., 2019). Therefore, the policymakers have to think seriously to transform public health into the best desirable state. For doing so, they have to take serious actions on a war footing (Wang, 2019; Shi and Zainuddin, 2020).

Moreover, recesses daily, the inclusion of indoor and outdoor curriculum, the development of strong control and monitoring mechanism, development and empowerment of the physical education professional teachers, support from the latest technological tools and equipment, social and cultural support for the after-school physical classes, and active parental support for the continuity of the PE are indispensable for the development of PE (Hastie et al., 2020; Shi and Zainuddin, 2020). Parents should be aware of the fact that it is more their responsibility than a school to take of the children for the holistic development of the learner. Furthermore, non-support after-school programs should include physical activity. In the same way, active transport to and from school can be a safe and effective way to increase students’ daily physical activity, especially where a large proportion of students live close to their school. Similarly, each community needs to examine systematically opportunities for community-based promotion of physical activity (Zhang, 2017). Likewise, inviting students’ families and other community members to participate in the physical training and sports events before- and after-school programs, including sports and active transport, will increase program sustainability to witness the progress of the student growth.

## Conclusion

In conclusion, the incorporation of the above-mentioned theories and models allows for the creation of better motor cycle and effective learning experiences. Therefore, to meet the demands of the ever-changing environment, China’s government has to adopt for cohesive, comprehensive, and applicable curriculum for implementation in less developed areas like Southern Ningxia. These are also cost-effective, socially and emotionally supportive; therefore, it is equally acceptable to teachers, parents, and students. Moreover, China’s people can develop a public–private partnership for the better implementation of TGfU, for PE to be an effective teaching strategy to ensure students are experiencing quality learning and desired aims, focus areas, and learning outcomes. Learning experiences designed in PE classes can be supported by constructivist approaches, inquiry-based learning, and the TGfU models through the use of pedagogical strategies, such as questioning, student-led activities, and group work. This article concludes by indicating that current and future teacher practitioners need to consider how to utilize educational theoretical models for supporting effective pedagogy in PE and undertake a concerted effort to create environments in which students take control of their learning and construct meaning relevant to their own lives—resulting in lifelong learning in PE.

## Recommendations

Mental and body growth and development, mutual compatibility, and consistency are the pre-requisites for balancing personality growth and, specifically, educational development. Historically, it is evident that human was engaged in physical and mental development. Indifferent eras and areas, ups and down can be seen in physical education, reasoned to many organizational, governmental, and environmental issues, which caused degradation of physical education and its development was ignored. This article presents a holistic view of physical education development. The author developed a model for uplifting physical education, integrating all possible and feasible content. Moreover, this manuscript also presents a historical overview of physical education, precise to China. Furthermore, it also recommends incorporating the latest technological tools like information and communication technologies and artificial intelligence for physical education development. Moreover, this article also emphasizes the cultural aspects, which work as blood and foundation for the development of physical education development. In the same way, it also proposes dynamic management and integration of the school management, professional teachers, local bodies, and government for inclusive physical education development.

This manuscript proposes a holistic framework for physical education development. In the ever-changing organizational environment, the needs and demands for physical education also change. This manuscript provides a continuous development model for the school, integrating education stakeholders. However, the study was limited only to the Ningxia province, and for getting more comprehensive and generalized results, the study may be incorporated into other regions and provinces of the country.

## Data availability statement

The raw data supporting the conclusions of this article will be made available by the authors, without undue reservation.

## Author contributions

XF: write up corrections, data curation, supervision, visualization, editing, and writing of draft. WZ: writing and software, conceptualization, methodology, review, and visualization.
