# Corrigendum: Dilemma and countermeasure of sustainable leadership in physical education development in southern rural Ningxia, China

**DOI:** 10.3389/fpsyg.2023.1172040

**Published:** 2023-05-12

**Authors:** Xiaoya Fu, Weiqiang Zhu

**Affiliations:** ^1^College of Physical Education and Health, East China Normal University, Shanghai, China; ^2^College of Physical Education, Ningxia Normal University, Guyuan, Ningxia, China

**Keywords:** sustainable leadership, sustainable strategies, sporting leadership, technological support, physical education

In the published article, there were errors in the affiliations of Xiaoya Fu and Weiqiang Zhu as published. Instead of “Xiaoya Fu^1^ and Weiqiang Zhu^2*^”, it should be “Xiaoya Fu^1,2^ and Weiqiang Zhu^1*^”.

In addition there was an error in affiliation 2 as published. Affiliation 2 was listed as: “Department of Education, Ningxia Normal University, Guyuan, China” but should be “College of Physical Education, Ningxia Normal University, Guyuan, Ningxia, China”.

In the published article, there was also an error in the caption of Figure 1 as published. “Status of non-availability of PE facilities (Li et al., 2018)” should have been “Status of non-availability of PE facilities (Source: Author's pilot data)”. The revised Figure 1 caption Figure 1 appears below.

**Figure 1:**
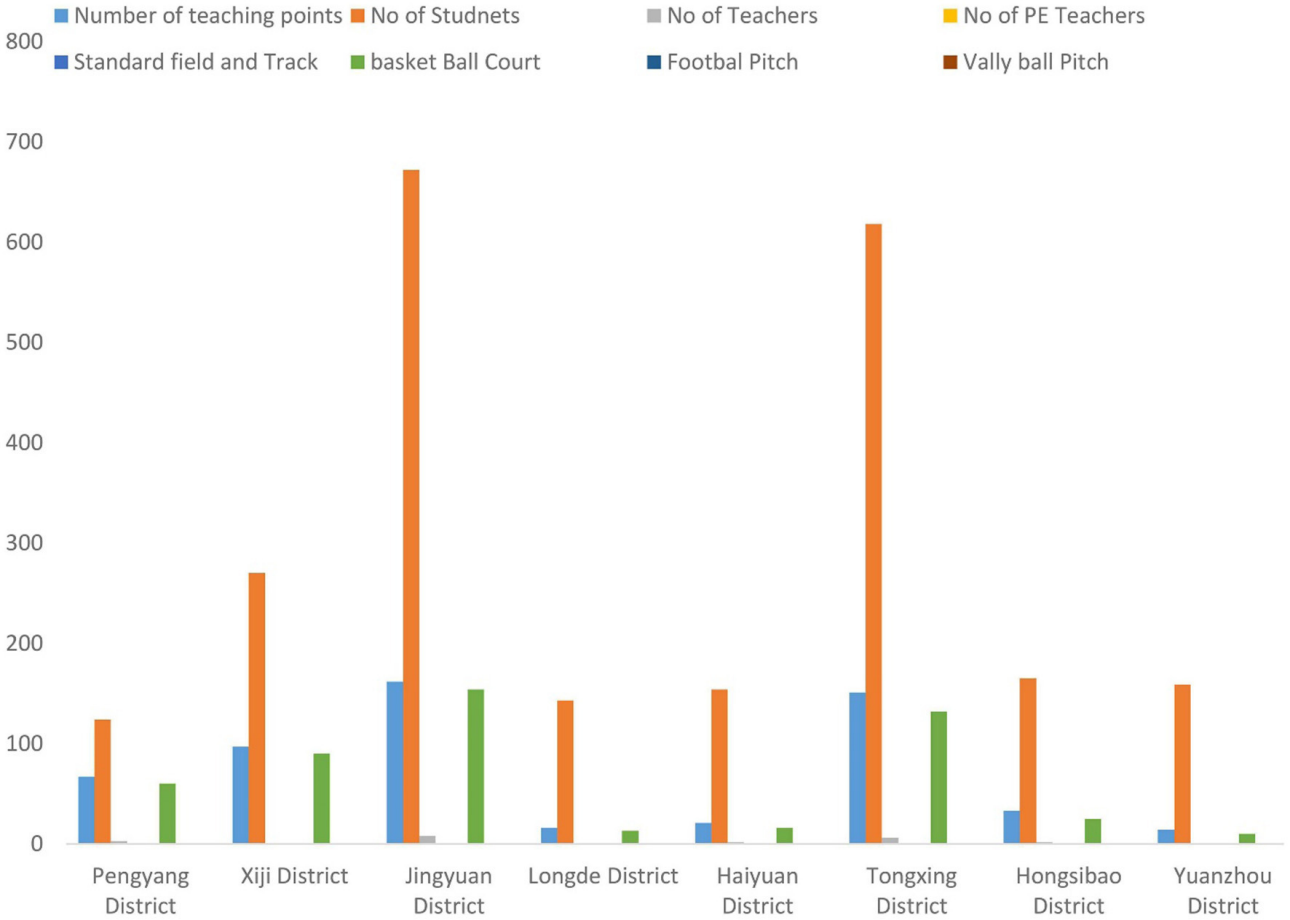
Status of non-availability of PE facilities (Source: author's pilot data).

In the published article, there was also an error in **Literature review**, “*Research design*”, Paragraph 1. In the second sentence the study location was given as “in the Sothern area of China” but should be “in the mountainous and rural areas of Southern NingXia”. The revised paragraph is included below.

“Based on the objectives of the study, the multi-stage mixed method was applied to explore and comprehend the underlying phenomena in detail. In the first stage as shown in Table 1, content analysis was done besides contextual analysis to get the ground information of the PE in the mountainous and rural areas of Southern NingXia. In the second phase, a pilot study was conducted to explore the underlying state of PE. Based on the pilot study results, a qualitative open-ended interview was conducted with the education experts to further clarify the stances raised in the pilot phase of the study; during the interview, the objective of the interview was explained verbally and consent was scored. In the final stage, from the reliability and generalizability standpoint, a quantitative, cross-sectional large-scale study was conducted. Data were collected from the two-state of southern areas of Southern Ningxia in China using the 5-Likert scale and adopted questionnaire added in Supplementary Appendix. Moreover, to make the result more cohesive, specific, and reliable, the target population was divided into two strata, i.e., Faculty (Teachers) and Students, to get both perspectives individually and clearly. Furthermore, a convenient sampling technique was applied in the third phase of the data collection, and the reason for it was that those qualify the requirement, i.e., enrolled in school as a student and teacher, were eligible for inclusion in the study”.

In addition, in **Literature review**, “*Qualitative interview thematic analysis*”, Paragraph 1. The study location was given as “in the Hubei city of Ningxia Province” but should be “in the mountainous and rural areas of Southern NingXia”. The corrected paragraph appears below:

“In the first phase, a qualitative study was conducted as per the need and requirements of the study design. The study was conducted in February 2019 in the mountainous and rural areas of Southern NingXia. An open-ended structure questionnaire was designed on the results of the pilot study and a total of 10 experts participated in the interview, among them six were men and four were women, having bachelor's degrees with professional (education) related experiences. The main question asked was “Do you consider that psychical education is declining in the remote areas of China?,” followed by several contextual supportive mini questions and explanations, to clarify the content clearer for understanding. The thematic analysis of the open-ended interview is given here”.

The authors apologize for these errors and state that this does not change the scientific conclusions of the article in any way. The original article has been updated.

