# Supplementary material for: Dilemma and countermeasure of sustainable leadership in physical education development in southern rural Ningxia, China
Source: Front Psychol. 2022 Aug 29;13:947694. doi: 10.3389/fpsyg.2022.947694 (PMC9465482; doi:10.3389/fpsyg.2022.947694)
Supplement: Supplementary file 1 [file Table_1.DOCX]

1: Strongly Disagree

2: Disagree

3: not to mention

4: Agree

4. Strongly Agree

Research questionnaire adopted for this study

| S. no | Questions | 1 | 2 | 3 | 4 | 5 |
| --- | --- | --- | --- | --- | --- | --- |
|  | **Availability of the Facilities** |  |  |  |  |  |
|  | Our school has good indoor game facilities. |  |  |  |  |  |
|  | Our school has good outdoor facilities. |  |  |  |  |  |
|  | Our school has good area for indoor games. |  |  |  |  |  |
| 4. | Our school has good outdoor facilities. |  |  |  |  |  |
| 5 | Our school has structured time table for physical education. |  |  |  |  |  |
|  |  |  |  |  |  |  |
|  | **Trained Professional Physical Teacher** |  |  |  |  |  |
| 6 | Our school has trained Physical education teachers. |  |  |  |  |  |
| 7 | Our school physical education teachers are professional trained. |  |  |  |  |  |
| 8 | Our school Physical education teachers is active in physical education classes. |  |  |  |  |  |
| 9 | Our school physical education teacher shows more interest in arranging sports events. |  |  |  |  |  |
| 10 | The PE teacher is the central figure in the formation process of the pupil. |  |  |  |  |  |
|  | **State Supports for Physical Education** |  |  |  |  |  |
| 11 | In our area, physical education is promoted by the state. |  |  |  |  |  |
| 12 | State has developed community level committees for physical education development. |  |  |  |  |  |
| 13 | State has developed structure program and policy guidelines for physical education. |  |  |  |  |  |
| 14 | Athletes are encouraged and recognized by the state. |  |  |  |  |  |
| 15 | It is necessary to maximize the conditional capacities in pupils. |  |  |  |  |  |
|  |  |  |  |  |  |  |
|  | **Support from school** |  |  |  |  |  |
| 16 | In our school, Physical education is valued by other teachers. |  |  |  |  |  |
| 17 | Other subject teacher gives extra-passing grades to the athletes. |  |  |  |  |  |
| 18 | Our school leadership supports Physical education. |  |  |  |  |  |
| 19 | Our management provide the required funds for sport events. |  |  |  |  |  |
| 20 | Our school management provide structured program for the physical education. |  |  |  |  |  |
|  |  |  |  |  |  |  |
|  |  |  |  |  |  |  |
|  | **Miscellaneous Problem of PE** |  |  |  |  |  |
| 21 | Crowded curriculum |  |  |  |  |  |
| 22 | Lack of facilities |  |  |  |  |  |
| 23 | Difficulty engaging students |  |  |  |  |  |
| 24 | Students have low level of interest in PE |  |  |  |  |  |
| 25 | Peer pressure |  |  |  |  |  |
| 26 | Physical education not priorities in the school |  |  |  |  |  |
| 27 | We Focus on too many traditional sports |  |  |  |  |  |
| 28 | Past negative experiences with Physical education |  |  |  |  |  |
| 29 | Large class sizes |  |  |  |  |  |
| 30 | The school environment does not encourage |  |  |  |  |  |
| 31 | Cost of subject |  |  |  |  |  |
| 32 | Staff use outdated teaching methods |  |  |  |  |  |
|  | **Student Related Problems** |  |  |  |  |  |
| 33 | Lack of student engagement |  |  |  |  |  |
| 34 | Expressed dislike for activity |  |  |  |  |  |
| 35 | Lack of intrinsic and extrinsic motivation |  |  |  |  |  |
| 36 | Intrapersonal barriers |  |  |  |  |  |
